# One-stage posterior debridement, autogenous spinous process bone graft and instrumentation for single segment lumbar pyogenic spondylitis

**DOI:** 10.1038/s41598-021-82695-2

**Published:** 2021-02-04

**Authors:** Bao Su, Ke Tang, Wei Liu, Xiaoji Luo, Zhengxue Quan, Dianming Jiang, Xiaohua Peng

**Affiliations:** 1 Department of Orthopedics, The First Affiliated Hospital of Chongqing Medical University, 1st Youyi Road, Chongqing, 400016 People’s Republic of China; 2 Department of Rehabilitation, The First Affiliated Hospital of Chongqing Medical University, 1st Youyi Road, Chongqing, 400016 People’s Republic of China

**Keywords:** Clinical trials, Pain, Bacterial infection

## Abstract

To compare the ﻿surgical outcomes of autogenous spinous process with iliac bone graft in managing single segment lumbar pyogenic spondylitis (PS) after posterior debridement and instrumentation. We performed a retrospective study for adult patients with single level lumbar PS. 60 patients with single segment lumbar PS underwent one-stage posterior debridement, autogenous bone graft and instrumentations. The patients were divided into Group A (autogenous iliac bone) and Group B (autogenous spinous process). Preoperative Charlson comorbidity index (CCI) was analyzed to assess﻿ the comorbidity. Low back pain was ﻿evaluated using the visual analog scale (VAS). Neurological status was ﻿assessed with the American Spinal Injury Association (ASIA) scale. Clinical infection index including the C-reactive protein (CRP) and erythrocyte sedimentation rate (ESR) was also reviewed. Moreover, fusion and changes of sagittal alignment were investigated radiologically. There was a significantly longer operative time, hospital stay and greater blood loss in group A. The VAS scores improved significantly at each follow-up interval and post-operative VAS score was significantly lower in group B. At the last follow-up, ESR and CRP returned to normal for all patients. There was at least one grade level improvement in ASIA score. No statistical difference in corrected rate, loss of sagittal angle and lumbar lordosis was found between the two groups. There was no significant difference in fusion rate, mean fusion time and complications between the two groups. Compared with iliac bone graft, the autogenous spinous process bone graft can be less invasive and painful for the single segment lumbar PS. One-stage posterior debridement, autogenous spinous process bone graft and instrumentation can provide satisfactory results for appropriate cases.

## Introduction

Pyogenic spondylitis (PS) encompasses a broad range of clinical entities, which is rare but severe, even potentially life-threatening. Although the pathophysiology of PS is not fully clear, it commonly arises from a hematogenous spread of bacteria. Therefore, conservative treatment with immobilization and systemic administration of antibiotics will be first recommended for patients with PS^1^. However, spinal instability, neurological compression or recurrent infection are deemed as potential surgical indications^2^.

The purpose of surgical treatment for PS is sufficient decompression and radical debridement, followed by an anterior fusion. After extensive debridement of the infected tissue, structural bone or cage grafting would be performed as structural grafts to repair the bone defects. However, conventional autologous bone graft is often limited due to extensive bone loss and may be complicated by donor site morbidity. Posterior approach is becoming more popular for dealing with anterior vertebral column pathologies including infection, which can stabilize the spine and correct kyphosis, even has a lower incidence of complications than anterior surgery^3^. In addition, we considered the autologous spinous process, which could be achieved in the same operation area with lower donor site morbidity and less invasion, can be used as an ideal interbody fusion material for PS with posterior surgery.

In summary, this study aimed to compare the clinical and imaging outcomes of PS treated by one-stage posterior debridement with autogenous spinous process or autologous iliac bone strut as structural grafts.

## Materials and methods

### Patient population

**Figure 1:**
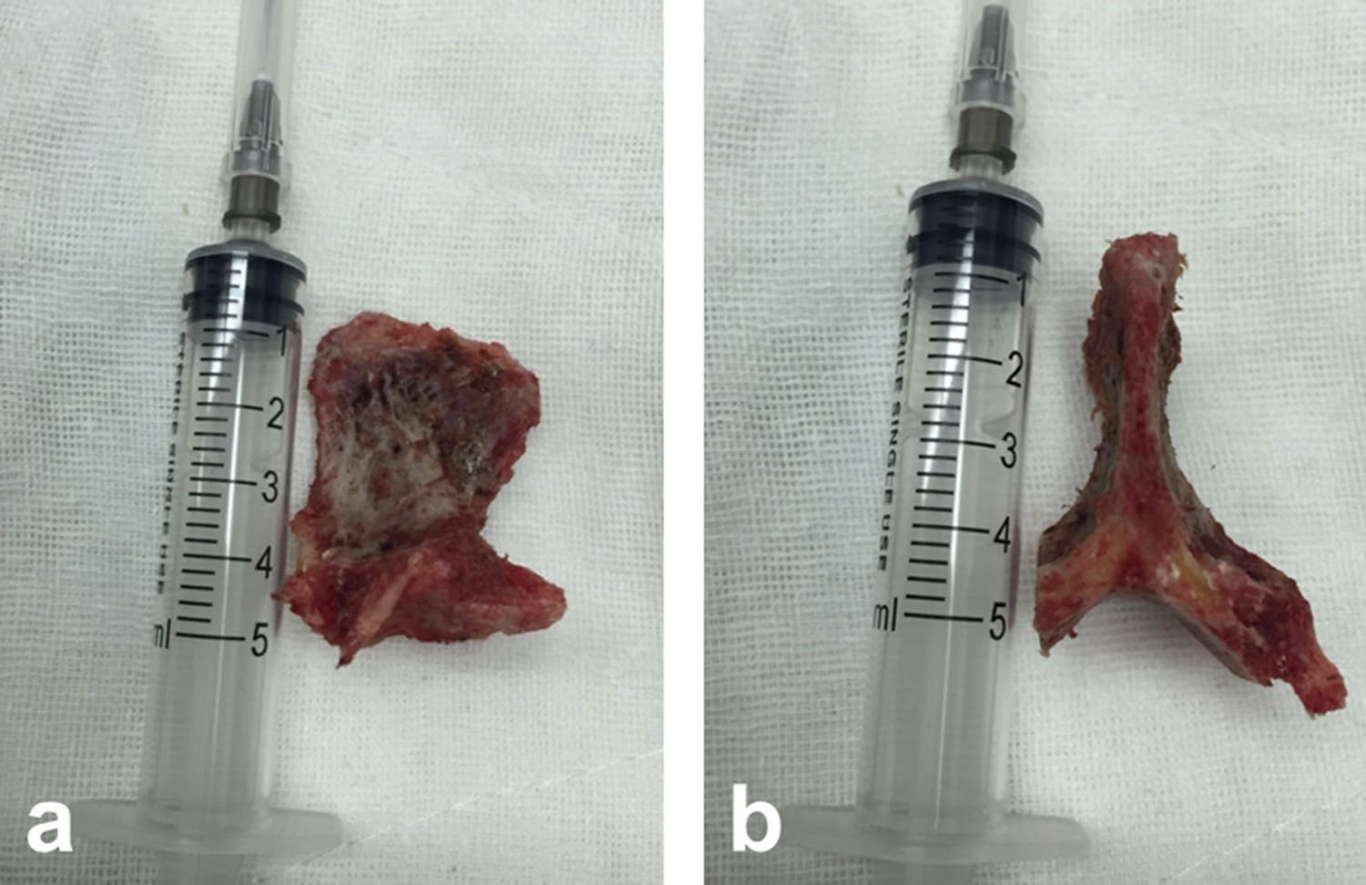
The specially formed autologous spinous process for interbody fusion obtained in the operation area (**a** A–P position, **b** lateral position). The cortex bone of the spinous process was grinded before grafting.

After obtaining written informed consent from all patients and ﻿the ethics approval from the Institutional Ethics Committee of ﻿the First Affiliated Hospital of Chongqing Medical University, this retrospective cohort study was conducted in one spine center. The study has been registered in Chinese Clinical Trial Registry on Dec 18, 2019. Clinical trial registration number: ChiCTR1900028301 (http://www.chictr.org.cn). All methods were carried out in accordance with relevant guidelines and regulations (Declaration of Helsinki). Between January 2011 and January 2016, 60 consecutive patients (24 men and 36 women) with single segment lumbar PS underwent one-stage posterior debridement, autogenous bone grafts fusion, and instrumentations in the study. In group A, 33 cases obtained autogenous iliac bone graft for reconstruction. In group B, 27 cases used previously obtained specially formed autologous spinous process (grind the cortex bone of the upper and lower surface) in the operation area for reconstruction (Fig. 1). The primary diagnosis was confirmed mainly according to medical history, ﻿radiologic findings (X-ray, computed tomography, magnetic resonance images), increased inflammatory indicators including white blood cell (WBC) counts, C-reactive protein (CRP) level and erythrocyte sedimentation rate (ESR). ﻿Charlson Comorbidity Index (CCI) scores were also calculated based on the past histories and laboratory data on admission. Indications for surgery include severe pain, unsensitive to antibiotics, obvious or imminent neurological dysfunction, epidural abscess, local kyphotic deformity. ﻿Cases were excluded if there was previously hardware placement, lumbar spine tuberculosis cases, or any decubitus ulcer at the time of diagnosis. The patients with more than two lumbar vertebral bodies involved were also excluded.

### Surgical technique

Briefly, after verifying the involved lumbar disc fluoroscopically, the posterior elements including spinous process, lamina and facet joints were exposed via a midline longitudinal incision (subperiosteally dissection) in prone position. Pedicle screws (Stryker Spine, Cestus, France) were implanted into two or four segments adjacent to the lesion segment. If the upper third of the vertebral body and pedicles were not affected, screws were also inserted into the ﻿diseased vertebra.

After performing semi-laminectomy or whole laminectomy of the decompression segment, we acquired the spinous process of the unaffected segment completely and cleared the necrotic tissues with curette and nucleus pulposus forceps. The affected intervertebral space was repeatedly rinsed with a large amount of normal saline and filled with gelfoam containing sensitive or empirical antibiotics (for cases with negative culture). Different methods were used for bone grafting: (1) Group A: obtained a tricortical iliac bone, trimmed to a suitable size, and implanted it into the bone defects. (2) Group B: previously obtained autologous spinous process was grinded the cortex bone and clipped into a suitable supporting bone graft. Then the autologous spinous process together with the bone granule were inserted into the anterior bone defects. When hemostasis and neural decompression were meticulously confirmed, the dura was covered with gelatin sponge, and a negative pressure drainage was placed before suturing the incision for each patient. The pus, granulomatous tissue and intervertebral disc were pathologically examined and cultured after operation.

### Postoperative care

After surgery, all the patients received initially ﻿4–6 weeks of intravenous antibiotics therapy (according to the antimicrobial susceptibility test results or empirical antibiotics treatment for cases with negative biopsy-culture), followed by oral antibiotics until CRP and ESR ﻿were normal.

### Clinical assessment

CRP and ESR values were chosen to evaluate the infection status. The visual analogue scale (VAS) score was used to assess low back pain and American Spinal Injury Association (ASIA) impairment scale was used to ﻿evaluate the neurological function before operation and during the follow-up periods.

Lumbar X-ray or 3D reconstruction CT images was performed for all patients 3 day, 3 months, 6 months, 12–30 months after surgery. The same two authors evaluated the fusion condition according to the images. Solid bone fusion was confirmed if there was continuous trabeculae between the graft and adjacent vertebral body without radiolucency, mottling, collapse of the graft, or motion beyond 3°^4^. The angle of lumbar lordosis was measured by the intersection of lines drawn parallel to the inferior endplate of T12 vertebrae and the superior endplate of S1 vertebrae. The Cobb angle formed between the upper endplate of the vertebra above the infected vertebra and the lower endplate of the vertebra below the infected vertebra in the sagittal plane was used to evaluate the correction effect of local fusion segment kyphosis.

The Student's t test, Mann–Whitney U test and ﻿Fisher’s exact test were used for statistical analysis with IBM SPSS software package, version 26.0 (IBM Corp., Armonk, USA). P < 0.05 was considered statistically significant.

## Results

### Clinical results

**Figure 2:**
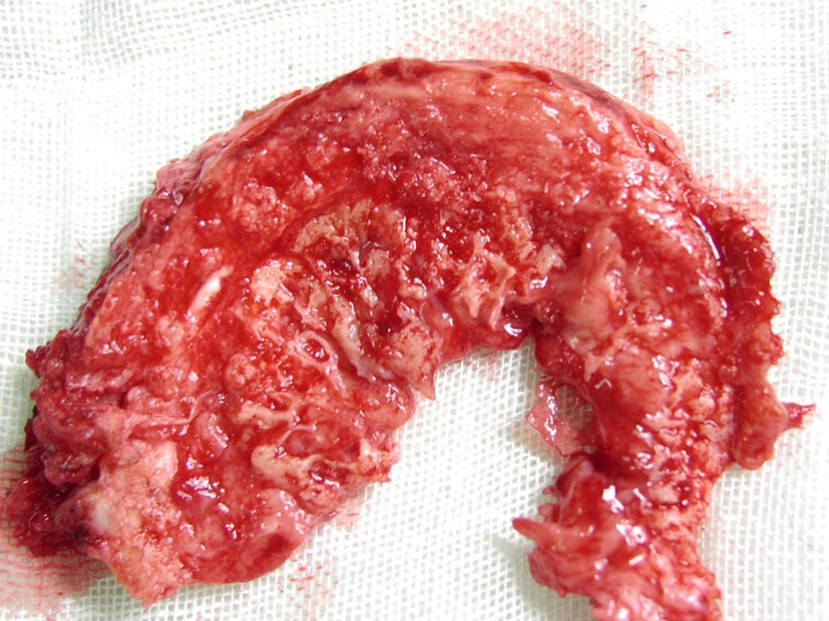
The necrotic intervertebral disc scraped off in the operation.

The vertebral levels involved in our patient population ranged from L1-2 to L5-S1. The average follow-up time was 20.5 ± 6.0 months in group A and 18.4 ± 4.1 months in group B (12–30 months, P > 0.05). Both groups had similar CCI distribution. No recurrence was observed during the whole follow-up. According to intraoperative findings, the intervertebral space is filled with necrotic intervertebral disc, abscess and sequestra. The necrotic intervertebral disc was fragile and easily scraped off (Fig. 2).

On admission, 11 patients in group A (33.3%) and 8 patients in group B (29.6%) suffered fever. The bacterial culture of the intraoperative specimen or blood was positive in 41 patients (group A: 22 patients; group B: 19 patients) including *Staphylococcus aureus* (22 cases), *Enterobacter cloacae* (7 cases), *Acinetobacter baumannii* (3 cases) and others (9 cases).

Complications including major vessel injury, dural tear, nerve root injury, and pulmonary embolism were not observed in the further follow-up. There was no abscess or fistula after removing the irrigation and drainage tubes.

Compared with Group A, the operative time and hospitalization duration were shorter, and the operative blood loss was less in group B (P < 0.05), however, no statistical difference between the two groups was found in preoperative CRP or ESR (P > 0.05). Levels of CRP and ESR in both treatment groups continued to decline during follow-up. Autologous iliac bone and spinous process graft had no effect on CRP or ESR levels. What’s more, the post-operative VAS scores of group B were lower (P < 0.05). While at the end of follow-up, the VAS score, CRP and ESR were all significantly improved in both groups (P < 0.05). The clinical results of the two groups were shown in Table 1 for comparation.

**Table 1:** Comparisons of clinical results between the two groups.

Clinical features	Group A (n = 33)	Group B (n = 27)	P value
**Infected spinal level**			
L1–2	1	1	–
L2–3	5	6	–
L3–4	9	7	–
L4–5	11	8	–
L5–S1	7	5	–
Charlson comorbidity index	1.8 ± 1.0	2.0 ± 1.1	0.501^a^
**VAS score**			
Pre-operative	6.9 ± 1.4	7.3 ± 1.3	0.455^b^
Post-operative	3.6 ± 1.1	2.6 ± 0.8	0.017^b^
Last follow-up	2.3 ± 0.9	2.1 ± 0.8	0.628^b^
Follow-up duration (mon)	20.5 ± 6.0	18.4 ± 4.1	0.334^b^
Operative duration (min)	231.2 ± 46.4	172.9 ± 42.7	0.004^b^
Operation blood loss (ml)	406.7 ± 87.8	308.3 ± 57.2	0.004^b^
Hospital stay (day)	29.7 ± 8.0	23.8 ± 5.2	0.046^b^
Fever	4 (33.3%)	3 (25%)	
**Mean value of pre-operation**			
ESR (mm/h)	85.2 ± 19.9	81.5 ± 22.3	0.676^b^
CRP (mg/l)	57.0 ± 35.8	48.2 ± 28.2	0.508^b^
**Mean duration of normalized ESR after operation (mon)**			
ESR	2.0 ± 0.6	2.3 ± 0.7	0.360^a^
CRP	1.7 ± 0.8	1.3 ± 0.8	0.225^a^

^a^Data was ﻿analyzed using Mann–Whitney U test.

^b^Data was ﻿analyzed using Student t test.

### Radiological changes

**Figure 3:**
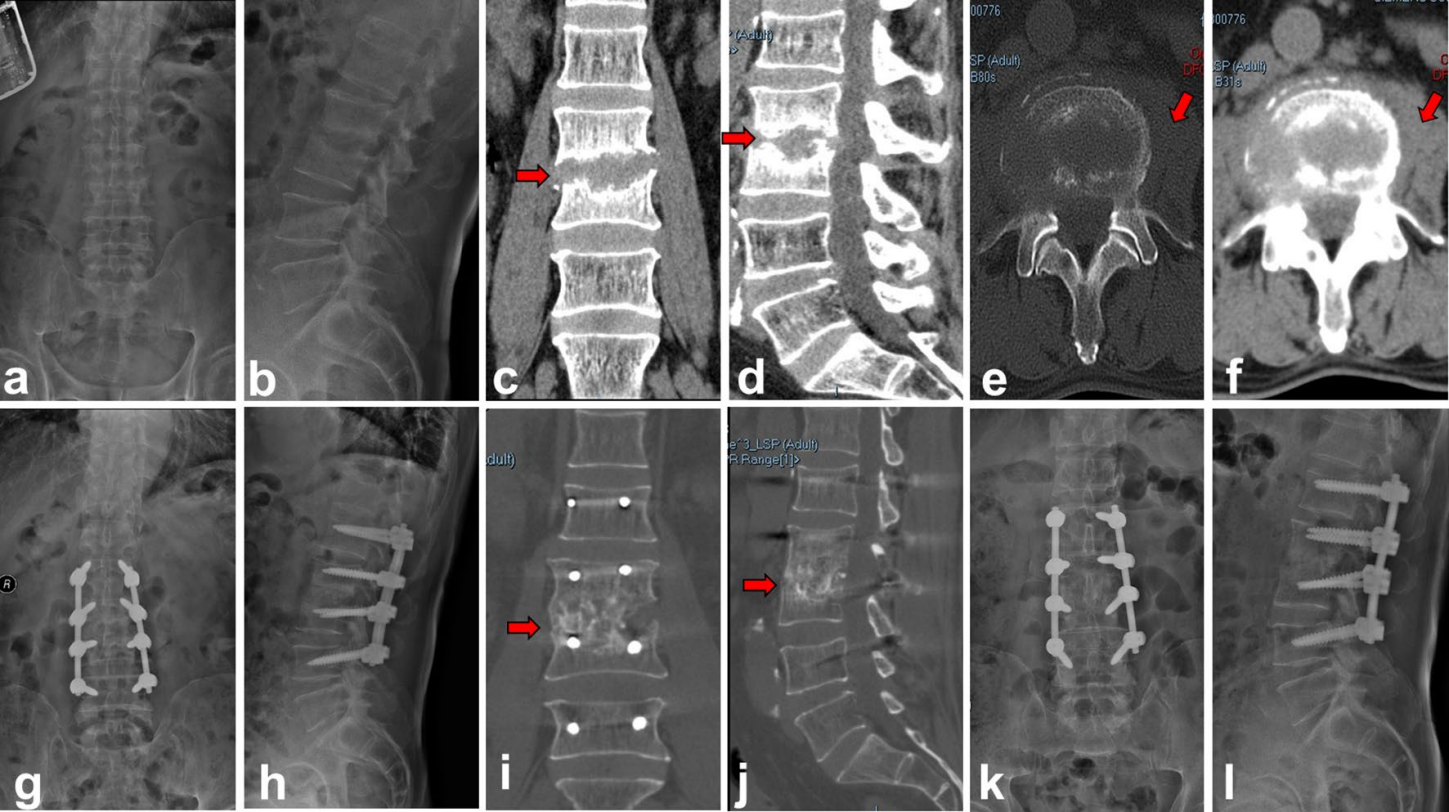
Autologous iliac bone group (group A). A 68-year-old male with L3–4 lumbar pyogenic spondylitis (**a**–**f**). Preoperative X-ray and CT showed that L3–4 vertebral body destruction with sequestrum formation, intervertebral space stenosis and psoas abscess (red arrow). (**g**,**h**) Postoperative X-ray showed iliac bone graft and internal fixations were in good location. (**i**–**l**) CT and X-ray taken at 6 months after operation showed solid bone fusion between L3 and L4.

**Figure 4:**
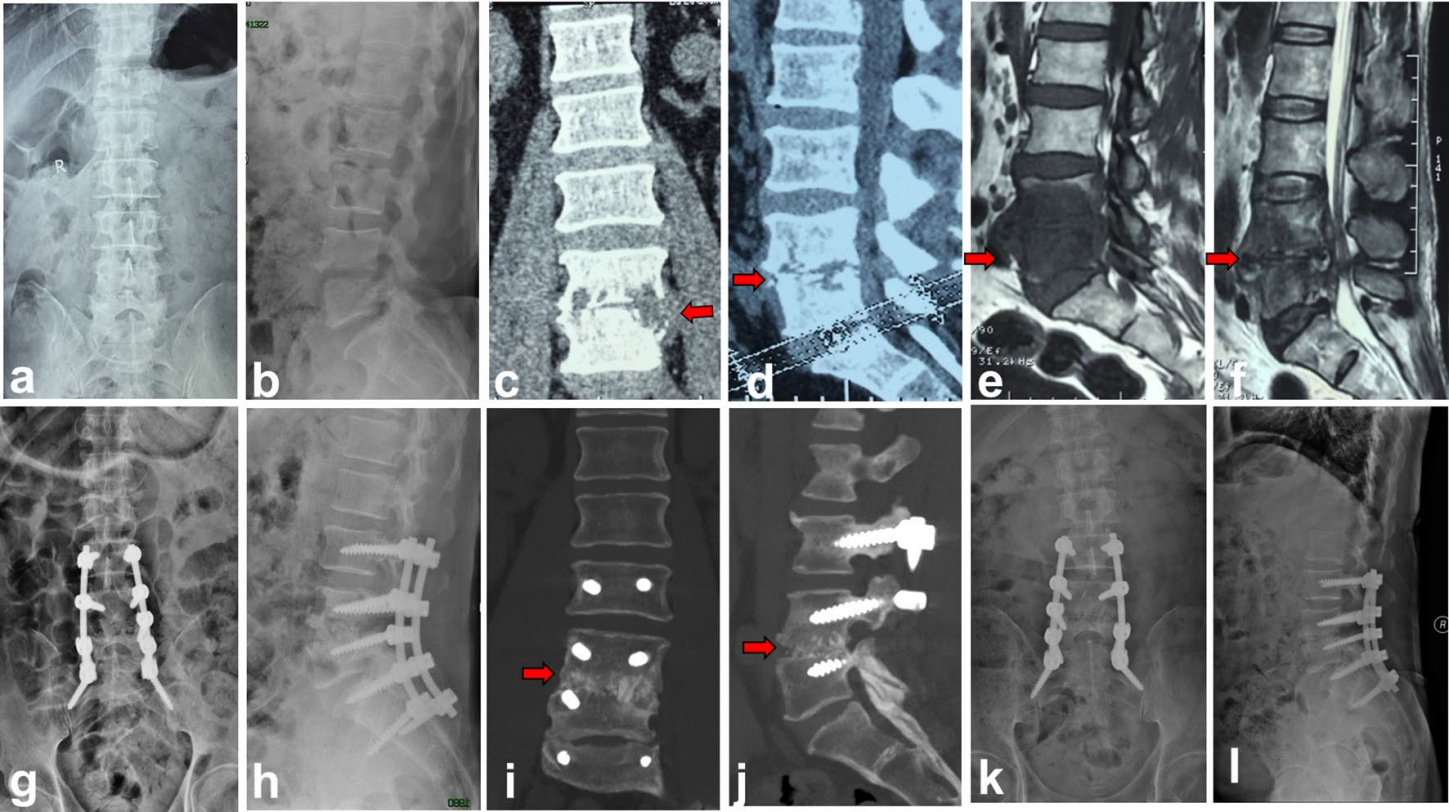
Autologous spinous process group (group B). A 62-year-old male with L4–5 lumbar pyogenic spondylitis. (**a**–**f**) Preoperative MRI and CT showed there were bone destruction, intervertebral space stenosis and massive paravertebral abscess (red arrow) at L4–5. (**g**,**h**) Postoperative X-ray showed autologous spinous process, screws and rods were in good location. (**i**–**l**) CT taken at 5 months after operation showed solid bone fusion between L4 and L5.

The comparison of the fusion rate, average fusion time, lumbar lordosis and sagittal angle results between group A and group B were summarized in Table 2 and representative radiological images of one patient in each group were shown in Figs. 3 and 4. The mean preoperative lumbar lordosis angles of group A and group B were 45.7° ± 8.9° and 44.1° ± 10.0°. These were corrected to 51.2° ± 7.2° (P < 0.05) and 52.6° ± 7.7° (P < 0.05) at the last follow-up, respectively. The mean preoperative sagittal Cobb angle of group A was 5.9° ± 3.3°. This was corrected to 14.1° ± 4.9° (P < 0.05) after the immediate operation. There was a mean loss of 4.6° in the follow-up period and the mean sagittal Cobb angle was 9.5° ± 3.2° at the last follow-up. In Group B, the mean preoperative sagittal Cobb angle was 6.1° ± 4.9°. This was improved to 15.5° ± 5.0° after immediate surgery and 11.5° ± 5.0° at the final follow-up (with a mean loss angle of 3.75°), indicating a significant correction. Compared with the preoperative values, the mean sagittal Cobb angles of group A and group B were significantly improved at the last follow-up.

**Table 2:** Comparison of Radiological changes between the two groups.

Clinical features	Group A (n = 33)	Group B (n = 27)	P value
**Sagittal angle (°)**			
Pre-operative	5.9 ± 3.3	6.1 ± 4.9	0.924^a^
Postoperative	14.1 ± 4.9	15.5 ± 5.0	0.612^a^
Last follow-up	9.5 ± 3.2	11.5 ± 5.0	0.290^a^
Correction loss	4.6 ± 3.7	3.75 ± 2.0	0.498^a^
**Lumbar lordosis (°)**			
Pre-operative	45.7 ± 8.9	44.1 ± 10.0	0.519^a^
Last follow-up	51.2 ± 7.2	52.6 ± 7.7	0.458^a^
**Bone graft fusion**			
Fusion rate	31/33 (93.9%)	24/27 (88.9%)	0.649^b^
Mean fusion time (month)	6.8 ± 1.4	7.9 ± 2.2	0.131^c^

^a^Data was ﻿analyzed using Student t test.

^b^Data was ﻿analyzed using ﻿Fisher’s exact test.

^c^Data was ﻿analyzed using Mann–Whitney U test.

The fusion rate in group A and group B were 93.9% and 88.9%. And the mean fusion time in group A and group B were 6.8 ± 1.4 (range 5–9) and 7.9 ± 2.2 (range 5–12) months, respectively. ﻿Two patients (6.1%, 2/33) diagnosed with ﻿methicillin-resistant S. aureus (MRSA) in group A experienced no fusion at 24-month follow-up. Those numbers come to 11.1% (3/27) in group B. The fusion rate and average fusion time have no difference between the two groups (P > 0.05).

### Neurological function

At the final follow-up, ASIA score of each patient had improved by at least one grade. ASIA grade in group A changed from C to D in 9 cases and from D to E in 24 cases. In group B, ASIA grade changed from C to D in 8 cases, from C to E in 2 cases, and from D to E in 17 cases. At the end of follow-up, there was no significant difference in ﻿postoperative neurological function between the two groups (Table 3).

**Table 3:** Comparison of neurological ﻿status between the two groups.

ASIA scale	Preoperative		Postoperative	
	Group A (n = 33)	Group B (n = 27)	Group A (n = 33)	Group B (n = 27)
A	0	0	0	0
B	0	0	0	0
C	9	10	0	0
D	24	17	9	8
E	0	0	24	19

## Discussion

Restoration of a stable anterior spinal column is essential for normal spinal biomechanics and infection healing. Therefore, bone grafting is a vital procedure in surgical treatment of PS, which is not only for bone fusion, but also for structural reconstruction. The present study is the first to report the efficacy of autologous spinous process used as bone graft in managing PS through posterior approach.

There are several surgical approaches for the treatment of PS, including anterior^5,6^, posterior^7,8^, and combined anterior–posterior approaches^9,10^. The choice of the appropriate surgical procedure is often determined on surgeons’ preference, neurological deficits and predicted spinal instability^11–13^. However, recent literature suggested that posterior only approach may be associated with fewer complications compared with the combined approach including anterior debridement and posterior instrumentation. By a posterior surgery, the spinal deformity can be corrected and the anterior column can be supported, which also helps to promote fusion^14^. In addition, the lumbar stenosis, which is a common disease in the aged, can be cured at the same time. Zhang et al. reported that single-stage posterior fusion was effective in the management of mono-segmental lumbar or lumbosacral PS, which was compatible with our findings in this study^15^.

Bone graft materials had a significant influence on fusion. The autologous bone grafting has been widely used in traditional surgery for PS. The iliac bone strut was the most commonly used graft due to its high ratio of bony fusion, which was considered as the “gold criteria”. Past studies have reported that the single-level lumbar fusion rate with iliac bone strut was as high as 90–100%^16^. Using local autograft as a substitute for iliac bone strut has theoretical inferiorities in that there exists a smaller volume and a relatively higher proportion of cortical to cancellous bone, which inevitably contains lower osteogenic cells and a lesser amount of trabecular area for performing the osteogenic and osteoconductive properties of autograft^17^. However, iliac bone grafting has obvious disadvantages of neurovascular injury, persistent pain and potential infection of the donor site^18^. Furthermore, because the majority patients with PS are elderly and suffer osteoporosis, there is a risk of hematoma and instability^19^.

Our study has revealed that use of a local bone graft technique (spinous process) showed the similar fusion rate and fusion time as that of iliac bone strut with less surgical time and fewer complications. Furthermore, we found the autologous spinous process bone graft has some advantages over iliac bone strut graft, which are embodied in the following aspects: (1) the surgeons have to spend extra time on some surgical procedures such as making another incision, harvesting iliac bone, hemostasis and suturing. However, in group B, they just need to removed cortical part of the spinous process to make bone grafts. What’s more, the decompression, debridement, deformity correction and intervertebral grafting and instruments can be performed simultaneously in one incision. Obviously, this will save surgical time and reduce operation time and blood loss. (2) Patients in whom tricortical full-thickness iliac graft was harvested had a quite high prevalence of donor site morbidity, such as persistent pain of the donor site which corresponds to our result that the post-operative VAS score was significantly higher in group A. However, using the autogenous spinous process grafting may relieve the postoperative pain, shorten the hospital stay and reduce hospitalization expense, which is in line with the concept of enhanced recovery after surgery. (3) Compared with iliac crest, autologous spinous process bone grafts also have a better supporting biomechanical properties and abundance sources. Multiple spinous process bone grafts can be taken according to the defects without increasing extra trauma.

In our study, we innovatively removed cortical part of the spinous process to make bone grafts, which could be performed simultaneously in one incision with debridement, decompression, deformity correction and intervertebral bone grafting and instrument just through the posterior only approach. More importantly, this study showed the fusion rate of spinous process bone graft was similar to that of iliac bone strut graft. This may be associated with the following reasons: (1) most of the cortical bone of the spinous process where contact with the end plates was removed before grafting. This may increase the fusion rate of the grafting. (2) With the firmly fixed instruments, spinous process bone grafts only need to bear little load, which is beneficial for the fusion between the graft and the vertebral body. (3) Besides the spinous process bone graft (structural graft), the bone granule (non-structural graft) harvested during posterior decompression were also packed into the intervertebral space as much as possible. Widening the graft area may be helpful for fusion. (4) The complete removal of all infected and necrotic tissue allows for extensive contact between the graft and adjacent vertebral cancellous bone, which may facilitate fusion.

The present study had several limitations. First, this study is a retrospective study with a small sample size. According to our results, there are statistical differences among the four indexes (P < 0.05): post-operative VAS score for low back pain, operative duration, operation blood loss and hospital stay. Using the mean value and standard deviation of the four indexes, ﻿powers (1-β error probability) and effect sizes of the four indexes calculated in the G-Power software were ﻿0.976, 0.998, 0.998 and 0.912 and 1.03, 1.30, 1.32 and 0.87 respectively. However, larger randomized control trials are needed to analyze the outcomes of autogenous spinous process bone graft in the setting of lumbar pyogenic spondylodiscitis. A prospective study with a more homogenous populations might have provided more rigid efficacies and limitations of this surgical strategy. Moreover, the case series was limited because we excluded patients who obtained allogeneic bone or cage insertion without bone grafting from the pelvis treatments. Whether the autogenous spinous process bone graft was more applicable than allogeneic bone or cage in the treatment of lumbar PS remained uncertain and should be studied in the future work.

In conclusion, compared with iliac bone graft, autologous spinous process bone grafting had similar fusion rate but less invasion for single segment of lumbar PS. These two grafting methods ﻿showed similar clinical outcomes for patients with an adjacent small abscess in the anterior spine and mild vertebral destruction, those with epidural abscesses around the dura, and lumbar canal stenosis. One-stage posterior debridement, autogenous spinous process bone graft and instrumentation can alleviate low back pain, correct kyphotic deformity and improve nerve function for proper cases.

## Trial registration

The study has been registered in Chinese Clinical Trial Registry. Clinical trial registration number: ChiCTR1900028301 (http://www.chictr.org.cn).
